# Books and Pamphlets Received

**Published:** 1867-09

**Authors:** 


### *Books and Pamphlets Received.*

*The Physiology and Pathology of the Mind.* By HENRY MAUDSLEY, M.D., Lond., etc., etc. New York: D. Appleton & Co., 443 & 445 Broodway. 1867. pp. 442.

A beautifully printed book, on tinted paper, in the publishers' best style. A work of profound thought and erudition, which we cordially commend to the careful perusal of all thinking and reading physicians.

*A Treatise on Human Physiology;* Designed for the use of Students and Practitioners of Medicine. By JOHN C. DALTON, M.D., etc., etc. Fourth Edition, revised and enlarged.

With two hundred and seventy-four Illustrations. Philadelphia: Henry C. Lea. 1867. pp. 695.

The present edition of this now standard work fully sustains the high reputation of its accomplished author. It is not merely a reprint, but has been faithfully revised and enriched by such additions as the progress of physiology has rendered desirable. Taken as a whole, it is unquestionably the most reliable and useful treatise on the subject that has been issued from the American press.

*Dr. McDowell's Letter to the Assembly of Teachers at Cincinnati, Ohio, 1867.* St. Louis: P. M. Pinckard & Co., Nos. 508 & 510 Pine St. 1867.

Pepper, salt, and mustard on excoriated backs. A pamphlet which proves that its eccentric author, although "chock-full" of quips, cranks, and prejudices, and particularly liable to "go off half-cocked," at all events has not a particle of humbug in his composition, nor will he put up with it in any wise. His own idea, that the action of the "Assembly of Teachers" was in the nature of a sectional attack on the South-West, is simply absurd. The majority of Northern and Western teachers entertain no such foolish prejudices as he attributes to them.

*Personally*, we look upon the proceedings of that "Assembly" as but a shallow device to galvanize sundry moribund schools into a semblance of vitality; to make more places for little men, anxious for position; and, particularly, to keep up the somewhat stale notoriety of sundry seedy parties who "live and move and have their being" (what there is of it) only whilst wriggling down to the footlights of some professional Trades-Union convocation.

Dear Bro. McDowell, the game is scarcely worth the candle.

Angular Curvature of the Spine. By Benjamin Lee, M. D., &c. An excellent brochure which we fervently recommend, as from the perusal of it, we have gained an unaccustomed comfort amid the dreamy platitudes with which we have been afflicted in times gone by. It is not an advertising dodge of some worthless though finical instrument, but a good sound little treatise.

Clinical Instruction at the Chicago Charitable Eye and Ear Infirmary.—During the winter courses of instruction at the medical colleges of Chicago, there will be regular clinical lectures on diseases of the eye at the Infirmary. This institution offers the medical student and practicing physician unusual opportunities for the practical study of diseases of the eye and of their medical and surgical treatment.

During the past year, *five hundred and fifty-five* patients were treated at the Infirmary, of which *eighty-seven* required important surgical operations. An aggregate of *3197* patients have received the benefits of the institution since its organization, nearly ten years since.

There has been an average daily attendance of 26 patients at the infirmary during the past three months.

Dr. Holmes will give special courses on the use of the ophthalmoscope.

The Legislature of the State of Illinois, at its last regular session, appropriated the sum of $10,000, for the support of the poor of the State, during treatment, at the Infirmary for diseases of the eye or ear.

The Legislature of the State of Wisconsin recently appropriated the sum of $500, for the support of Wisconsin soldiers receiving treatment at the Infirmary for diseases of the eye, contracted in the army during the late war.

*Trustees*—W. L. Newberry, President; P. Carpenter, Vice-President; S. Stone, Secretary; E. B. McCagg, Treasurer; W. H. Brown, William Barry, T. B. Bryan, C. G. Hammond, E. C. Larned, Wesley Munger, E. W. Blatchford, Daniel Goodwin, Jr.

*Consulting Surgeons*—Prof. J. W. Freer, M.D., Prof. H. A. Johnson, M.D.

*Attending Surgeons*—E. L. Holmes, M.D., Prof. E. Powell, M.D.

*Superintendent*—G. Davenport.

*Matron*—Mrs. Davenport.

